# Vibrational Spectroscopy Fingerprinting in Medicine: from Molecular to Clinical Practice

**DOI:** 10.3390/ma12182884

**Published:** 2019-09-06

**Authors:** Vera Balan, Cosmin-Teodor Mihai, Florina-Daniela Cojocaru, Cristina-Mariana Uritu, Gianina Dodi, Doru Botezat, Ioannis Gardikiotis

**Affiliations:** 1 Faculty of Medical Bioengineering, Grigore T. Popa University of Medicine and Pharmacy of Iași, Iași 700115, Romania; 2 Advanced Centre for Research-Development in Experimental Medicine, Grigore T. Popa University of Medicine and Pharmacy of Iași, Iași 700115, Romania (C.-T.M.) (F.-D.C.) (C.-M.U.) (D.B.) (I.G.).

**Keywords:** fourier transform infrared spectroscopy, Raman spectroscopy, ex vivo, in vivo, clinic, fingerprint

## Abstract

In the last two decades, Fourier Transform Infrared (FTIR) and Raman spectroscopies turn out to be valuable tools, capable of providing fingerprint-type information on the composition and structural conformation of specific molecular species. Vibrational spectroscopy’s multiple features, namely highly sensitive to changes at the molecular level, noninvasive, nondestructive, reagent-free, and waste-free analysis, illustrate the potential in biomedical field. In light of this, the current work features recent data and major trends in spectroscopic analyses going from in vivo measurements up to ex vivo extracted and processed materials. The ability to offer insights into the structural variations underpinning pathogenesis of diseases could provide a platform for disease diagnosis and therapy effectiveness evaluation as a future standard clinical tool.

## 1. Introduction

The origins of infrared (IR) spectroscopy date back to 1800, when the astronomer William Herschel discovered the infrared region of the electromagnetic spectrum positioned beyond the red limit of the visible band [1]. Vibrational spectroscopy provides structural information in both qualitative and quantitative analyses of practically any compound. The principle of IR spectroscopy is established on the interaction of the chemical bonds of a sample with the radiation of a light source to generate a signature fingerprint in the form of a spectrum.

In the last decade, the increased development in both instrumentation and chemometric methods positioned the IR qualitative and quantitative analytical technique as the most widespread method for determining the chemical structures of molecules. In IR spectroscopy, the route to the measurement solution can be a journey with interdependent steps, starting from a high-quality instrument with the correct configuration, the right sampling, preset calibrations, standard operating procedures, analytical methods, measurements, software platform, data processing, and validation. In the following subsections, we will present the “recipe” for successful implementation of an IR measurement.

### 1.1. IR Multi-Range Options

**Figure 1:**
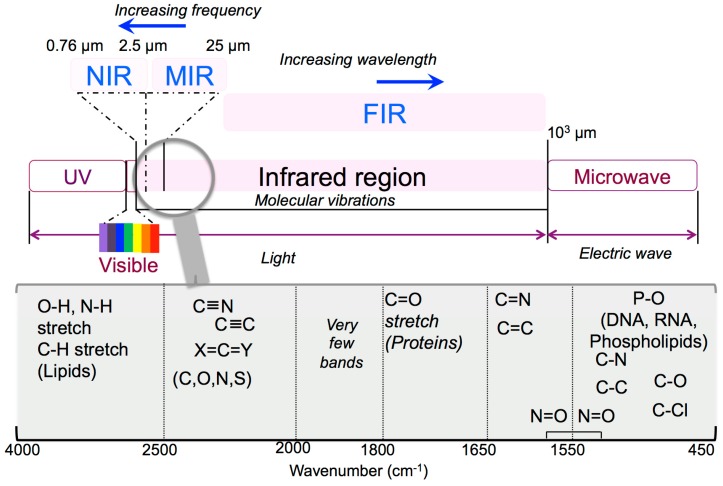
Infrared multi-range options.

A good spectrometer is essential but not the whole solution. Analytical infrared spectra are centred on the absorption or reflection of the electromagnetic radiation detected among 1 and 1000 μm and divided in three forms of IR: near IR (NIR) in the 0.76–2.5 μm region, mid IR (MIR) in the 2.5–25 μm region, and far IR (FIR) beyond 25 μm (Figure 1).

NIR spectroscopy (12,500 − 4000 cm^−1^) is a useful measurement of the manufacturing process but is particularly useful for raw materials checking and verification. MIR is a well-recognized and reliable method through which different compounds can be identified and quantified. The 4000 − 400 cm^−1^ MIR area is the most commonly used infrared region for biological applications, which includes the so-called fingerprint regions representative for lipids, proteins, amide I/II, carbohydrates, and nucleic acids (Figure 1). More insights on these two powerful analytical tools can be found in Reich’s book chapter [2] that focuses on modern pharmaceutical MIR and NIR applications starting from basic principles of both techniques, sampling of solid drug molecules and excipients, and up to data processing. FIR spectroscopy (400 − 20 cm^−1^) provides information on the highly ordered structures such as fibrillar formation and protein dynamics [3], since it is more sensitive to the vibrations from the peptide skeletons and hydrogen bonds than MIR [4].

Further information on IR spectroscopy theory related to factors that control absorption, band shapes in liquids, calculation of vibrational spectra, and vibration rotation spectra can be found in Steele’s book chapter republished in 2017 [5].

Discovered in 1928 by C.V. Raman, who detected scattered light with a different wavelength to the original, Raman spectroscopy provides fingerprint-type information on the composition and arrangement of defined molecules and material identification [6]. The laser monochromatic light that stimulates the quantification of Raman effect [7] is directed onto the cell where photons interact with the sample and energy can be both vanished (Stokes) or expanded (anti-Stokes) [8]. Then, the scattered photon excites a particular molecular vibration and produces a Raman spectrum where each molecule leaves a specific pattern, according to the corresponding functional group [9,10]. Raman spectroscopy detects the modifications in the conformation and constitutes of cells, tissues, deoxyribonucleic acid (DNA), proteins, and lipids [11,12]. Complementing infrared spectroscopy, the Raman technique possesses special advantages, including being nondestructive; fast to acquire; capable of providing information at the molecular level and analyzing samples in aqueous solutions since water produces a weak Raman scattering; and important in the biochemical field where researchers study the ionization behavior, pH change, or amino acid configuration. According to Ramirez and Gonzales [13], the main disadvantages of Raman results from the weak signal and the presence in biological samples of an intense fluorescence background noise.

Vibrational microspectroscopy associates infrared or Raman with microscopy and computer science for high-quality spectra in the diffraction limit analysis [14] by determining the chemical composition at single cell or subcellular levels [15]. The primary achievement of this technique is the spatial resolution that allows, besides a microanalysis on small samples, a spatially resolved localized chemical evaluation of the morphology (histology) of the structure [16]. Vibrational microspectroscopy found applications in biology and medicine as a diagnostic tool to discriminate control from unhealthy cell and tissues [17] due to its distinctive fingerprinting potential and the ability to identify changes that occur during normal cell cycle, necrosis, or apoptosis.

### 1.2. Sample Preparation

One of the assets of IR spectroscopy is its capacity to obtain spectra from different solids, liquids, and gases. In traditional transmission mode, the solids sample preparation involves grinding of the material to a fine powder followed by dispersion into a liquid matrix (mineral oil- nujol) to form a mull or in potassium bromide (KBr). Liquids are usually analysed as thin customised films with chosen thickness or pathlength formed between two IR transparent cells and a spacer. Different drawbacks were found in transmission sampling such as inevitable reproducibility issues and difficulties in sample preparation related to matrix ratios and homogeneity. The Attenuated Total Reflectance (ATR) technique deals with these concerns by providing high-quality spectra combined with enhanced reproducibility, since it is the probably the best IR sampling technique to our knowledge. ATR measures the changes that occur in a totally internally reflected infrared beam when the beam comes into contact with a sample through the surface of a crystal made of zinc selenide, germanium, and diamond. The sample is intact and unmodified since no other components are needed; therefore, ATR provides valuable data that cannot be obtained with any other method [18]. For biological materials, the most frequently used sample processing methods are formalin-fixed paraffin embedded material and fixed or unfixed cryopreserved frozen tissue, sectioned using a cryomicrotome [19].

### 1.3. Band Assignments

**Figure 2:**
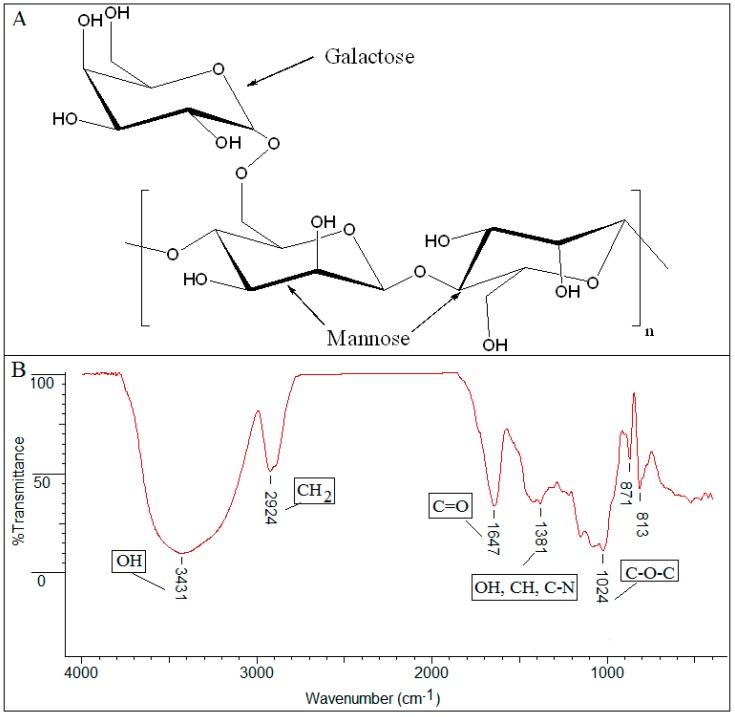
(**A**) Guar gum chemical formula; (**B**) FTIR-ATR spectrum of dried sample guar gum analysed using a Nexus FTIR Diamond instrument (Thermo Scientific) with a Smart Orbit diamond crystal ATR accessory in the range of 400–4000 cm^−1^.

Infrared spectra are “fingerprints” of the comprised molecules. A typical infrared spectrum as presented in Figure 2, is a ratio of sample spectrum and air spectrum (background). The X-axis (peak position) represents the frequency of a vibration of a specific part of molecule (4000 − 400 cm^−1^), and the Y-axis (peak intensity) informs about the absorbed sample energy (Transmittance (%T) or Absorbance). The spectrum [20,21] from Figure 2 representing a natural polysaccharide, presented as an example, can be interpreted as follows:

- the prominent peak in the region of 3431 cm^−1^ denotes the presence of a significant quantity of hydroxyl groups (OH) in the structure;
- the 2924 cm^−1^ peak is attributed to vibration of axial deformation of C–H of the CH_2_ group;
- 1647 cm^−1^ stretching suggests the presence of carbonyl of non-substituted amide and water;
- the 1381 cm^−1^ band corresponds to deformation modes with participation of the OH, CH, and C–N groups;
- the sharp peak at 1024 cm^−1^ is dominated by absorptions from the hydroxylic C–O single bond stretching of the C–O–C group in the anhydroglucose ring;
- the absorption bands between 871 − 813 cm^−1^ were attributed to the galactose and mannose moieties specific to guar gum.

It is well known that biological samples are essentially complex, comprised from a fusion of different proteins, lipids, nucleic acids, and carbohydrates; therefore, the infrared spectrum is the result of their characteristic absorption bands, explained in following subsections. Briefly, as presented by Caine at al. review [19], the bands in the 3050 − 2800 cm^−1^ range are controlled by the antisymmetric and symmetric C–H stretches, the ester carbonyl band from 1745 − 1725 cm^−1^ are specific for lipids, the absorption bands appearing in the 1700 − 1500 cm^−1^ region are defined by amide I and II groups responsible for the peptide linkages in proteins with a minor protein band often referred as amide III within 1350 and 1200 cm^−1^, and the antisymmetric and symmetric C−O and P−O areas (1235 − 1080 cm^−1^) are detected in DNA, ribonucleic acid (RNA) and phospholipids. Carbohydrate vibrations represented by glucose, fructose, and glycogen are positioned in the 1025–1150 cm^−1^ region.

The accurate assignment of the obtained stretching vibrations to the characteristic moiety is dependent on the pure sample spectra from international databases, along with other peaks in the spectrum. Any alteration in biological systems induced by a pathological condition produces significant structural and functional changes that are directly reflected in the vibrational spectra.

### 1.4. International Available Databases

Along with the development of FTIR and Raman spectrometers, there has been enormous increase for qualitative and quantitative chemical measurements through standardized methods. Therefore, if a specialist or nonspecialist looks at the infrared spectrum, they can estimate what functional groups are present and can even establish the chemical composition by comparison with known spectra. This widely used fingerprint approach is done by spectral search function usually available in the instrument software and has the capability of comparing thousands of spectrum curves and of suggesting the most similar spectrum. The identification is successful only if the same spectrum is part of the library compared with. 

Currently, there are multiples commercial reference spectra databases for both FTIR in ATR or transmission modes and Raman spectroscopy, collecting thousands of infrared spectra in digital format. These libraries are a comprehensive collection of spectra of pure compounds supplied by highly respected companies or institutions giving the user total confidence in the results obtained. One of the largest infrared spectra library providers is Spectral database for organic compounds- SDBS FTIR Transmission databases that includes more than 21,140 spectra, followed by Aldrich Database with 18,513 ATR FTIR spectra, IChem database with 12,706 ATR FTIR spectra, Elsevier FT-Raman and FTIR polymer database, National Institute of Standards and Technology (NIST) IR database, etc. The databases include different types of components including forensics, pharmaceuticals, polymer and polymer additives, solvents, biochemical, aldehydes and ketones, alcohols and phenols, and so on. The election of a suitable library depends on the application, instrument accessories, analysis conditions, and sample preparation.

### 1.5. Data Processing

Nowadays, the full-featured instrument control and data management software allows the user to easily acquire and process data, starting from sample identification and quantitative analysis to advanced applications. The software platform includes the required standard functions for infrared analyses: the apparatus control; ordinate modes; cm^−1^, nm and micron abscissa modes, blank acquisition; spectrum smooth; baseline correction; normalization; deconvolution; data processing; 1st–4th derivative with a variable filter; difference; interpolate; Kramers-Kronig; peak table; peak height and peak areas; validation; and additional optional utilities in order to offer advanced abilities designed for specific application areas.

### 1.6. Computational Methods and Chemometrics

An important aspect for spectroscopy users, besides the spectra itself, is the quantification and classification of components in a sample using more than one variable, through multivariate analysis techniques [22]. Given the molecular complexity of biological samples, several common techniques such as chemometrics that combine mathematical and statistical procedures are used to provide chemo-physical evidence from spectroscopic data [13]. According to the literature data, different multivariate data analysis techniques exist nowadays, divided into unsupervised and supervised methods, dependent on the objective of the analysis.

Traditional chemometric techniques include the following:

- Principal component analysis (PCA), the most basic feature extraction unsupervised techniques, based on the analysis of the variance of features within the full spectrum;
- Independent component analysis (ICA) that identifies spectral components by searching for independent components;
- Vertex component analysis (VCA) that is specifically designed for hyperspectral images;
- Partial least squares (PLS), the most widely used supervised multivariate data analysis technique that estimates and quantify components in a sample;
- Clustering unsupervised methods, used to identify biological subtypes within a sample, such as hierarchical cluster analysis (HCA), k-nearest neighbours (KNN), artificial neural networks (ANN), discriminant analysis (DA), and support vector machines (SVM).

More insights on chemometrics techniques can be found easily in the Encyclopedia of Spectroscopy and Spectrometry republished in 2017 [22].

### 1.7. Strategy in Biomedical Analysis

Without overseeing the virtues of other spectroscopic modalities, it is clear that vibrational spectroscopy has gained a certain place within the spectroscopic arsenal used to investigate biological materials.

Vibrational spectroscopy techniques were, are, and will be further exploited to analyse biological molecules due to their certain advantages [23], as described below:

- experimental accessibility to a number of infrared and Raman active transitions derived from specific moieties in spatially localized regions within the biomolecules;
- noninvasive method that does not involve spin labels or fluorescent probes;
- no limits on sample molecular weight, such as DNA;
- instantaneous snapshots of all molecular conformations;
- absence of line broadening compared with magnetic resonance spectra, due to relaxation phenomena;
- minimal sample preparation as described above;
- simplicity, rapidity, and low-cost;
- high molecular sensitivity joined with spatial resolution down to a few micrometers.

**Figure 3:**
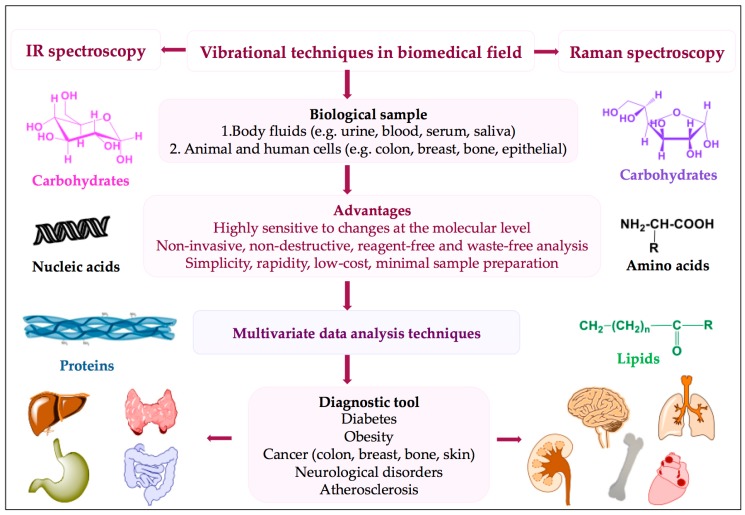
Strategy in biomedical applications.

The most important outcome of the IR technique is represented by its favourably sensitive assessment of the entire “-omics” [24] of a biological sample, which empowers the detection of molecular changes that may reflect early diagnosis and effective disease prognosis (Figure 3).

Given the powerful capacity of vibrational spectroscopy for detecting specific spectral components, this technique confronts also several usual drawbacks that can be grouped into the following: instrument fault (noise or artefacts), method liability coming from incorrect sampling technique, or uses of unsuitable reference methods or calibration files and operator errors, all producing the same outcome, namely inaccurate results.

Over the years, a number of well-recognized reviews have highlighted numerous features of vibrational spectroscopy in medical diagnostics and drug design, briefly detailed below in order to design the background and evidence our paper complementarity. In 2009, Carter et al. [25] pointed out the benefits of vibrational spectroscopic microprobes use for cells and tissues mapping and imaging. The authors provide only an application example, namely the infrared imaging of a cribriform ductal carcinoma in situ, outlining the essential need of multivariate statistical analyses for a direct tissue classification. Petibois and Desbat [26] compared in 2010 the main analytical performance metrics of current imaging methods used in the biological sciences and concluded that FTIR imaging systems still have several drawbacks such as inadequate lateral resolution, poor sensitivity, and inappropriate data treatments, features mandatory for clinical imaging. However, the authors anticipated that the detectors development would place FTIR spectroscopy and imaging as the future clinical instrumentations. Kazarian and Chan [27] summarized the ATR-FTIR spectroscopic imaging potential in biological systems applications. The authors gave a summary of the imaging fields of view, spatial resolution, and capabilities of various ATR imaging approaches currently available along with important details for simple sample preparation for in situ measurements, spatial resolution improvements, scattering reduction, dispersion of refractive index, variable depth of penetration, and contact pressure on biological tissues. The “Outlook for the near future” section expands the idea of integrating the ATR-FTIR imaging system with a planar, chip-based microfluidic device for the chemical analysis of cells and cell sorting, forensic science, body fluids, etc. The preliminary results obtained by the authors shown the path to design a microfluidic device that permits the fluid to stop within the image area for approximately 1 s. In 2014, the impressive work of Baker at al. [28] focused more on the protocol itself for collecting IR spectra and images from biological samples including the instrumental options available, sample preparation, different sampling modes, spectral data acquisition, data processing, quality control, spectral preprocessing, feature extraction, and multivariate data processing possibility than of the application and data correlation for diagnostic or prevention purposes. Therefore, the primary aim of this systematic review is to provide a complementary summary of vibrational spectroscopy biomedical applications for in vivo, ex vivo, and clinical diagnoses and to demonstrate the potential of this tool to be applied in current clinical practice.

## 2. Drifting from Molecular to Clinical Practice

Raman spectroscopy and FTIR are two noninvasive optical techniques that give valuable information on the chemical composition based on functional groups detection and spectral analysis of the obtained “fingerprints”.

According to the literature data, up to now, vibrational systems were used in medicine for cancer diagnoses, such as skin, breast, cervical, prostate, and gastrointestinal tumours; neurological disorders; diabetes; atherosclerosis; malaria-infected red blood cells; and monitoring of osteoarthritis and rheumatoid arthritis on cellular, animal, or clinical models that will be further discussed below.

### 2.1. Body Fluids

Taking into account the abovementioned features, both FTIR and Raman techniques were employed as diagnostic tools of different body fluids: serum [29], tears [30], saliva and urine [31], amniotic fluid (for the fetal lung maturity assessment), whole blood (for glucose analysis), synovial fluid (for arthritis diagnosis), semen, and vaginal secretions [32]. These vibrational procedures are less invasive than traditional biopsies, for example, in cancer screening, and at the same time are highly specific and able to perceive small deviations in the protein content during different stages of disorders progression.

As previously mentioned above, biological samples include in their composition lipids, proteins, sugars, and DNA [33], and any variation formed in the content or in the structure can be detected using IR [34]. For a detailed overview devoted to the common clinical analyses performed by IR spectroscopy of biological fluids, readers are asked to see Shaw and Mantsch on vibrational spectroscopy [35]**.**

Blood serum analysis and especially proteins analysis by vibrational spectroscopic methods can deliver essential information on patient condition and indicate the existence of different pathologies [36]. Both the MIR and NIR spectra of blood serum allows identification of the most abundant organic species (total protein, glucose, triglycerides, urea, albumin, and cholesterol). Blood serum includes nearly 20,000 distinctive proteins with 1 mM total concentration. The “peptidome”, known as the low molecular weight fraction of the serum, is present in relatively small concentrations, but it has a cancer-specific diagnostic data potential [37] mainly due to the fingerprint capability of the molecular events from different organs or tissues associated with cancer presence.

Over the years, numerous studies based on vibrational spectroscopy compared healthy serum with different types of cancer serum from patients, such as cervical [38], lung [39], leukemia [40], prostate [41], oral cavity [42], ovarian [43], and breast, and also analysed the drug efficacy during chemotherapy [44]. For example, the results showed that the main spectral modification between healthy and breast cancer patients were in the CH stretching vibrations area, the C–O ribose and its backbone, and P–O vibrations [45]. In the case of gastric cancer, the peak height ratio 2959/2931 could represent the differentiation standard from healthy patients.

FTIR spectroscopy, linked with PCA and linear discriminant analysis (LDA), is an innovative technique that explores the serum characteristics in breast cancer. In a report achieved on 86 from breast cancer and healthy women, serum samples were analysed by FTIR followed by PCA-LDA of the spectral data. The results indicated significant dissimilarities between the two groups in the following regions: 3700 − 3090 cm^−1^ (NH stretching), 3000 − 2800cm^−1^ (–CH_2_ and –CH_3_), 1760 − 1710 cm^−1^ (ester), 1710 − 1475 cm^−1^ (protein), 1350 − 1190cm^−1^ (collagen), and 1200 − 950 cm^−1^ (sugar). The major spectral difference in serum samples was related to protein conformation alterations, suggesting that FTIR and multivariate data analysis successfully discriminate the breast cancer serum [46].

The advantages for using IR-based methods in the hematology laboratory are detailed below:

- chemicals or specific molecular probes free;
- identification and quantification based on IR “spectral patterns” of the compounds;
- minimum sample quantities (μL of fluids or nearly 10^3^ cells);
- automation capability since IR systems can yield test results within minutes (≈15), with basic training of the operator.

In this context, vibrational spectroscopy was employed for the determination of hemoglobin (Hb) oxy-deoxy conversion in erythrocytes under stretching conditions [47] and biochemical parameters in human serum [48].

Immunoglobulin G (IgG) blood concentration is associated with humoral immunity level; therefore, abnormal IgG values are frequently considered to be a disease indicator or as an infection predisposition marker. According to available data, ATR-IR spectroscopy is typically used to measure IgG concentrations in human serum samples [49]. Similarly, ATR-IR combined with chemometrics successfully recognised serum from HIV-infected patients when compared to healthy controls with visible differences under therapy [50].

The fetal lung maturity can be anticipated by analysing two parameters, namely the lecithin/sphingomyelin ratio and the surfactant/protein ratio [51] using IR spectra of dry amniotic fluid films meanwhile saliva dried film MIR spectrum exposes not only the protein components but also thiocyanate (SCN–). At the same time, IR spectral features of saliva can determine complex biochemical profiles suitable to identify potential diabetes development indicators and to detect elucidate disease modes of action, risk factors related to diabetic complications, and therapeutic efficacy markers.

Likewise, the synovial fluid absorption patterns in the 2400 − 2000 nm region [52] could become the innovative differentiated diagnostic indicators for osteoarthritis, spondyloarthropathy, and rheumatoid arthritis. An encountered drawback of IR quantification is the low concentration of several target analytes (e.g., serum creatinine) that will be surmounted by “laminar fluid diffusion interface” preprocessing with microfluidics [53].

If we take a look at the literature reviews published in the field [33], it could be observed that biological and medical applications have progressed significantly in recent years. However, several downsides remain still an issue, namely the best sampling mode and the optimum sample preparation in order to minimize the serum preparation effects onto the spectrum [54].

Prior to IR spectroscopy translation in clinic, there are several issues that need understanding [55], such as the strong IR activity of water. The analysis of aqueous solutions in the transmission MIR mode faces an evident barrier given by the difficulty to drain and refill cells repeatedly in such a short pathlength. This obstacle has been finessed in two ways. The first and the best identified solution to minimize this obstacle is to use ATR spectroscopy, known to measure MIR spectra of strongly absorbing aqueous solutions, without the inconvenience and imprecision of the required short pathlengths. The second resolution is the water removal from the sample with the formation of a film by drying of the 5–50 µL spread liquid on a suitable substrate [56].

The use of Raman spectroscopy to analyse biological compounds has several advantages as follows: it requires a small amount of sample, it is a fast and resilient to water interference, it is noninvasive to the tissues, and it permits in situ detection. Raman can be employed to determine the proteins secondary structure, the interactions between anticancer drugs and DNA, and the diagnostic of injured tissues and cells and to analysis of body fluids from humans or experimental animals. For instance, in diabetic blood serum, Raman peaks were attributed to proteins, skeletal C–C stretch of lipids acyl chains, carbohydrates, and collagen [57]. In the same pathology, glucose, α-amylase, and ghrelin appetite hormone were identified by spectral analysis, suggesting their potential as individual salivary biomarkers for diabetes [58]. Spectral analysis of saliva evidenced several modifications in the proteins, lipids, glucose, thiocyanate, and carboxylate major metabolic components from healthy and diseased subjects. An interesting application of both techniques, vibrational and Surface-Enhanced Raman Spectroscopy (SERS), was the determination of drugs abuse such as diazepam, cocaine, cotinine, methamphetamine, and benzoylecgonine from oral fluids [59].

According to recent scientific data, biofluid assessments as diagnostic indicators poses numerous key benefits, translated in enhanced accessibility, repeated sampling procedure, and noninvasiveness, that could be applied in routine health analysis, blood intraoperative monitoring, or therapy. However, a more specialised vibrational technique, namely drop-coated deposition Raman spectroscopy (DCDRS), is needed to pre-concentrate the proteins from body fluids for an accurate analysis, for example, human tears evaluation by DCDRS indicated that both local and systemic disease biomarkers must be measured [60].

The concentrations of blood glucose measured by Raman spectroscopy evidenced results in good correlation with the reference values [61] as presented in Table 1. The same method detected molecular changes in the erythrocyte membranes [62] with the aim to diagnose type II diabetes by identifying lipid alterations (diminished liquidity and distorted phospholipid conformation).

Taleb et al. [63] used Raman technique to distinguish cirrhotic patients with and without hepatocellular carcinoma by analysing blood serum. A more recent study found that the neoplastic lymphocytes exhibited strong proteins characteristic band (1447 cm^−1^ and 1126 cm^−1^) but minor DNA peaks (1098 cm^−1^, 785 cm^−1^) when compared to normal cells [64]. Likewise, the Raman spectra of malignant and bladder cells denoted a superior concentration of proteins and nucleic acids in the bladder cells as in cancer cells [65]. Furthermore, the advanced confocal Raman microscopy and SERS offered phenotypic identification of white blood cells designed to develop innovative diagnostic kits for haematological tumours [66].

Raman spectroscopy was also used to identify and quantify the C-reactive protein (CRP) concentration in blood plasma [67], a sensitive biomarker of inflammation caused by bacterial infection, typically determined by time-consuming and high-priced immunoassays techniques. Bergholt et al. [67] showed that PLS analysis quantified CRP in blood serum samples obtained from 40 patients with a value of the root mean square error of cross validation of 10.8 mg/L. Also, PLS regressions were employed to quantify the fibrinogen concentration in blood plasma [68] along with the heparin concentration in patients’ blood throughout surgery [69].

Modifications of red blood cells infected with malaria [70] were intensively studied by Raman spectroscopy. Meanwhile, Hobro et al. [71] monitored the changes occurred in plasma, following malaria disease progression over 7 days and reported that the Raman peaks related to Hb and hemozoin denoted variations from the first day of infection while changes in erythrocyte membranes occurred around the fourth day, suggesting their suitability as malaria indicators.

In the last years, plentiful reports highlighted the effectiveness of vibrational spectroscopy techniques in the biochemical distorted tissues and summarized their experiences in atherosclerosis research [72,73,74,75]. Comprehensive overviews reported that the abovementioned analysis joined with other complementary procedures, such as atomic force microscopy (AFM) and confocal microscopy, could deliver high resolution images of the tissue morphological configuration; identify significant plaque structures, vascular wall constituents, endothelium dysfunction, and heart valve stenosis indicators; and moreover, follow the disease mechanism evolution related to heart valves pathological modifications and calcifications. The proposed techniques along with suitable data categorisation algorithms delivered a powerful tool for biochemical variations analysis of in vivo processed tissues or either diet–induced alterations or mixture of genetic and nutrition outcomes [76,77,78].

FTIR allows the identifications of lipids from the atherosclerosis plaque [79], proteins, and changes in proteins conformation, especially shifts of the proteins secondary structure, indicated by the intensification of β-sheet related configuration and also by the diminution of helical and unassigned conformations in atherosclerotic murine tissues [80] and even calcification detection (Table 1).

Taken into consideration the abovementioned features of FTIR and Raman hybrid systems, it is clear that Raman microscopy benefits from a better spatial resolution than FTIR [74], being effectively used on several plaque features evaluation such as collagen fibbers, fibrous cap, smooth muscle cells, internal elastic lamina, adventitial fat, ceroid, necrotic core/foam cells, intraplaque haemorrhage, carotene crystals, plaque surface thrombus, and calcification [73,74,81]. If we analyse a lipid fraction Raman spectrum, we could observe the following characteristic bands: 2885 cm^−1^ (C–H stretching), 1740 cm^−1^ (C=O stretching), 1674 cm^−1^ (C=C stretching mode) typical for cholesteryl esters and cholesterol, 1443 cm^−1^ (C–H bending), and 704 cm^−1^ (steroid rings) [82]. At the same time, if we use Raman confocal imaging, valuable tissue composition data could be obtained directly from the site location (e.g., cholesterol crystals and lipid droplets) and identify major variations of the unsaturation degree of lipids with submicron resolution [74].

The spectrum of pathologically damaged muscle of tunica media exhibits the main Raman peaks for proteins at 1660 cm^−1^ (amide I), 1244 cm^−1^ (amide III), and 1004 cm^−1^ (phenylalanine); meanwhile, thrombotic plaques (contain Hb) can be simply distinguished by the high intensity bands detected at 1580, 1130, 964, and 750 cm^−1^ assigned to Heme and calcium deposits. More insights on the plaque components Raman spectra related to elastin, collagen, Hb, cholesteryl esters and crystals, calcium salts, or ascorbic acid can be found in the literature [73,74,81]. The variations at the molecular level on atherosclerotic endothelial dysfunction were studied by Raman methodology based on the quantification of the tyrosine-phenylalanine content ratio in the endothelium [83]. At the same time, hydroxyapatite, the main calcification component of the atherosclerotic plaques, presents an intense Raman signal and can be identified through its characteristics bands around 1080 − 1070 and 964 cm^−1^, appointed to the stretching vibrations of the carbonate and phosphate groups, respectively [73,74]. While it was demonstrated that hydroxyapatite gives a strong Raman signal, several IR specific peaks were also depicted at 3400, 1100 − 1080, and 600 cm^−1^.

### 2.2. Animal and Human Cells

The live cell screening potential of Raman spectroscopy is based on the ability of the Raman effect to identify the chemical structures of a cell (lipids, proteins, and DNA) due to their specific vibrational spectra. Also, being based on vibrational spectra is not mandatory to label or stain the cells prior to imaging and, since water gives a weak signal, cells can be visualized in medium, conditions that assembled the normal physiological parameters [84,85].

The IR spectroscopy use should not be ignored in the cell live imaging, since it is also noninvasive and label-free and identifies the chemical structure as Raman spectroscopy [86]. However, we cannot underestimate the strong absorbing signal of water in infrared, which definitely restricts the analysis of samples in aqueous solutions. Therefore, Raman remains the standard solution over IR spectroscopy due to the ability to preserve the cells under physiological conditions and to achieve a better spatial resolution [6]. These features stimulated the development of Raman spectroscopy and imaging biomedical applications especially as in vivo or diagnosis tools [8]. However, the vast application of vibrational spectroscopy biomedicine faces the same issues, namely clinical translation that has a slow ongoing route, probably due to several drawbacks: regulatory aspects, standardization, and of course extensive clinical trials demands [55].

The exclusive features of vibrational analytical systems, namely molecular specificity and high optical resolution (confocal Raman microscopy) for 3D subcellular profiling, propose to be used in biomolecular alterations in vitro screening [87].

Since the first subcellular resolution evidence of FTIR microspectroscopy (FTIR-MSP) on single cells [88] in 1998, the interest for the use of IR spectroscopy for cellular diagnosis has grown markedly, achieving lately the best suitable spatial resolutions for single cells analysis [85,89].

The FTIR-MSP system analysed prostate cancer cell lines from different spots, paraffin embedded Gleason-graded malignant prostate, and benign prostate tissues. The peak area intensity ratios at 1080 and 1030 cm^−1^ allocated to phosphate and glycogen could be used to differentiate benign from malignant cells (higher ratio value for benign and lower for malignant ones). A valuable pioneered achievement was the separation of the different prostate metastatic cell lines by using FTIR coupled with PCA [90].

Schubert et al. [91] successfully differentiated squamous cytologically similar cells from healthy samples, low-grade squamous intraepithelial lesion (LSIL) and high-risk HPV (hrHPV) cells by infrared microspectroscopy (IR-MSP) combined to PCA, denoted as spectral cytopathology (SCP). The most notable spectral changes observed were in the 1670 − 1610 and 1550 − 1500 cm^−1^ protein amide I and II related regions, probably due to increased viral assembly and cellular degradation of proteins. Variations of the DNA and RNA areas were observed and explained by the viral genome high replication rate. The report demonstrated that SCP could be successfully used to distinguish abnormalities on morphologically undifferentiable cervical cells [91].

Kyriakidou et al. [92] showed that the high intensity of 3062 cm^−1^ band was correlated with cancer development, since the majority of proteins were amide B shaped and with β-sheet structure, by using FTIR spectroscopy and different human basal cell carcinoma, malignant melanoma, and nevus biopsies and compared with control normal skin tissue. In melanoma and nevus, the stretching vibration bands at 2950 − 2850 cm^−1^ related to CH_2_ were more intense; meanwhile, in basal cell carcinoma (BCC) the bands were less pronounced due to the amplified lipophilic nature as reported by the authors. The intensity of the 1744 cm^−1^ newly detected aldehyde peak assigned was boosted in melanoma and nevus, while in the spectra of normal skin, it appeared as a shoulder. The presence of both α-helix and random coil protein arrangements in melanoma and nevus determined the split of the 1650 cm^−1^ amide I absorption band into two, 1650 and 1633 cm^−1^. Also, the 1550 cm^−1^ amide II peak was shifted to lower frequencies at 1540 cm^−1^ for melanoma and 1536 cm^−1^ for BCC, denoting a potential loss of proteins native structure. The presence of B-DNA and Z-DNA bands at 841 and 815 cm^−1^ suggested that only the Z-DNA form is more pronounced in melanoma, whereas both DNA forms were detected in BCC [92].

The label-free potential along with the similar optical microscope spatial resolution, nondestructive nature, and rapid monitoring of Raman were beneficial for the cells and organelles molecular composition and cellular condition evaluation [93,94].

Oshima et al. [95] identified normal and four different types of human lung cancer cells cultured in dishes by Raman spectroscopy, LDA, PCA and an excitation laser at 532 nm. The Raman spectra of the RERF-LC-MS adenocarcinoma line, RERF-LC-MA squamous cell carcinoma line Lu-65 large cell carcinoma, EBC-1 squamous cell carcinoma line, and MRC-5 normal cell line revealed in principal a strong intensity at 1659 cm^−^^1^ peak endorsed to the protein amide I and the lipid C=C moiety. The spectral absorption bands detected were as follows:

- 1449, 1257, 1003, and 936 cm^−1^ were attributed to the CH_2_ bend, amide III, protein phenylalanine symmetric ring, and C–C unfolding;
- both weak and sharp Raman bands at 1618, 1605, 1209, 1175, 852, 642, and 620 cm^−1^ were assigned to tryptophan, tyrosine, phenylalanine, and aromatic amino acid deposits;
- 1577, 1421, 1340, 1086, 830, 785, and 720 cm^−1^ were related to DNA and RNA content; and
- intense peaks at 1583, 1127, and 747 cm^−1^ were associated with cytochrome c (cyt-c) in a reduced state.

As denoted by PCA and LDA, cyt-c existence and used as independent variables contributed to the discrimination of cancer normal cells but also to the histological type of the original cancer and malignancy nature, namely squamous cell carcinoma, adenocarcinoma, and small and large cell carcinoma.

In 2015, Talari et al. [96] analyzed the Raman differentiation between breast cancer cell lines (MCF-7 and MDA-MB-436) and normal breast MCF-10A through dispersive Raman spectroscopy due to the difference in biochemical compounds namely proteins, nucleic acids, and lipids. The cancer cells presented high lipidic and proteinic information in the 3050 − 2800 cm^−1^ and 1800 − 500 cm^−1^ regions. The lipids (2882 cm^−1^) and proteins (2940, 2921 and 2948 cm^−1^) vibrations and protein-to-lipid ratio separated MCF-7 line from the cancer cells. In the case of amide III, the identification was possible (through lipids, proteins, and nucleic and amino acid) in the range of 1380 and 1190 cm^−1^. After LDA, the prediction between a normal breast cell line and two lines of breast cancer had a value of 91% specificity and 100% sensitivity, combining Raman spectroscopy and chemometrics.

An important contribution to the cell cycle changes and cancer evolution is represented by the monitorization of lipid metabolism. In this sense, Chaturvedi et al. [97] used Raman-MSP to evaluate the biomolecular cascade events related to the conversion of a normal cell into an invasive breast cancer cell under physiological settings. The two analysed spectrum regions namely 3000 − 2800 cm^−1^ and 1800 − 700 cm^−1^ evidenced an amplified lipid amount for the invasive cells when compared with normal ones, as follows: immortalized cells exhibited also intensified bands allocated to symmetric CH_2_ stretching, and the converted cells displayed intensified peaks attributed to CH_3_ groups of both proteins and lipids. The same pattern of high lipid content was observed for the highly invasive breast epithelial cells (HMLE-Twist, MDAMB231) when compared with noninvasive epithelial cells (T47D, BT-474, HMLE); the data was sustained by the fluorescence staining tests with BODIPY and Nile Red biochemical assays. The multivariate analysis (PCA-LDA mode) of the Raman spectral data supported the foundation of a cluster cell type dependency model with an elevated level of sensitivity.

The differentiation between normal breast cell (MCF-10A) and breast cancer cell lines (MDA-MB-453, MDA-MB-231) was achieved by Lee et al. [98] using three techniques: Raman spectroscopy coupled with PCA, AFM, and optical microscopy. The evaluation of the obtained Raman spectra of both cell types revealed obvious changes in the regions related to amides I at 1650 cm^−1^, CH_2_ bending in the 1500 − 1450 cm^−1^ range, CH_2_ wagging/twisting at 1300 cm^−1^, amide III at 1250 cm^−1^, phenylalanine at 1000 cm^−1^, and S–S bonding vibration at 500 cm^−1^. The Raman-PCA results matched with the AFM morphology and optical transmission intensity evidenced the significant differences at intracellular level.

Intracellular localization of doxorubicin (DOX) by Raman-MSP coupled with confocal microscopy was achieved using A549 lung cancer cell line [87]. The authors highlighted by PCA spectral variations (before and after DOX) the interaction of cancer cells with DOX. The results showed that Raman-MSP detected DOX inside the cells and elucidated the local biomolecular variations produced by the drug. Further analyses are considered to be performed in order to evidence the potential of Raman spectroscopy to elucidate the drug action and response mechanisms at the molecular level, such as the early apoptotic effect in the nuclear areas with subcellular resolution [87].

The drug design development requires, besides the fundamental preclinical studies, important steps related to cytotoxicity and biological outcomes at the molecular level along with the mechanisms that underline the process. Batista de Carvalho et al. [99] monitored the cellular effect of Pt_(II)_ and Pd_(II)_ dinuclear chelates with spermine (Pt_2_Spm and Pd_2_Spm) with cisplatin (cis-Pt(NH_3_)_2_Cl_2_) drug (concentrations of 2–8 μM) in a human triple-negative metastatic breast cancer cell line (MDA-MB-231) by Raman synchrotron-radiation IR-MSP after 48 h exposure. The PCA data denoted a clear dependence of drug concentration and differentiation between control and drug-treated cells and between mononuclear vs. polynuclear and Pt_(II)_ vs. Pd_(II)_. The identified drug action spectral biomarkers were based on variations of proteins, lipids, and DNA peaks. The authors observed that the Pd_(II)_ chelate pointed the proteins while its Pt_(II)_ homologue disturbed the cellular lipids, revealing the different cytotoxicity incidence pathways. Overall, the developed studies established the noninvasive potential of Raman and FTIR-MSP to acquire “unique spectral signatures” of the molecular and biochemical interaction effects of cells and anticancer active compounds, as revealed by changes of particular intracellular constituents.

In 2017, a reorganized drug mixture of bezafibrate and medroxyprogesterone acetate (BaP) was tested in vitro and in vivo and the results revealed anti-leukaemic activity but without elucidating the action pathway [100]. ATR-FTIR, synchrotron radiation FTIR, and Raman-MSP were used to overcome this drawback by identification of the two AML cell lines biochemical structure in the presence and absence of BaP aiming to expand the clinical value. The main spectral dissimilarities evidenced on single living, dehydrated, and fixed cells exposed significant variations only on the cellular lipid composition with drug therapy with no impact on DNA. It is important to mention that, according to the authors, this reaction is independent by cell apoptosis; therefore, the unusual therapy mixture primarily focuses on lipids that activate specific signalling routes [100].

Using confocal Raman-MSP and immortalized human A549 lung cancer cells, MRC5 fibroblasts and three bronchial epithelial human cell lines (HBEC), Surmacki et al. [101] delimited efficiently the immortalized cell lines based on lipid composition dissimilarities located at 1440, 1301, and 1264  cm^−1^ and on DNA content at 784 cm^−1^. The chemometric PLS-DA model analysed all the high relevance Raman bands of all five cell types, revealing the following details: proteins were located at 1674, 1658, 1580, 1239, 1166/1174, 1003, and 641 cm^−1^; lipids were at 1658, 1440, 1301, 1264, and 718 cm^−1^; nucleic acids were at 1458, 1316, 828, and 784  cm^−1^; and carbohydrates were at 1085, 1043, 944, and 881 cm^−1^, assuring high values for sensitivity (96.3%) and specificity (95.2%).

The Raman technique linked with multivariate statistical analysis was employed for stem cells and their derivatives identification and separation. Chan et al. [102] identified and classified pluripotent human embryonic stem cells (hESCs), human fetal left ventricular cardiomyocytes (FLV-CMs), and hESC-derived CMs (hESC-CMs) according to their biochemical nature with 96%, 98%, and 66% accuracy values, respectively. The Raman spectra of the analysed cells evidenced the following characteristics:

- FLV-CMs vs. hESCs: lower peak intensities for 1578, 1320, 1128, 1090, 854, 811, and 785 cm^−1^ and slightly superior intensity of the 937 cm^−1^ region;
- hESCs vs. FLV-CMs: higher content of DNA and RNA according to the 1090 cm^−1^ (PO_2_^−^ stretch of the DNA phosphate backbone), 937 cm^−1^ (protein α-helix carbon backbone stretch), 811 cm^−1^ (RNA O-P-O stretching), and 785 cm^−1^ (DNA cytosine ring) peaks;
- hESCs vs. hESC-CMs: similar pattern regarding the DNA/RNA bands with lower intensity at 1320, 1090, 811, and 785 cm^−1^ and different trend for proteins, lipids, and carbohydrates located in the 1450 − 1320 cm^−1^ and 980 − 930 regions. The abovementioned results could represent the foundation for establishing a label-free noninvasive automated approach for hESC*s* and CMs discrimination valuable in cell-based heart therapies.

Human-induced pluripotent stem cells (hiPSCs) were characterized and compared with hESCs and differentiated hESCs by Tan et al. [103] using Raman spectral bands in the 1073 − 687 cm^−1^ region collected from living cells for 20 days. Similar spectral pattern was observed for hiPSCs and hESCs but distinguishable from differentiated hESCs, with variations sustained by the PCA.

Overall, Raman-MSP potential as a noninvasive tool in cancer diagnosis has been demonstrated. By adding the confocal microscopy capability, the optical resolution feature was enhanced, allowing the analysis of the biochemical processes at subcellular level and, therefore, making the system an ideal in vitro screening tool of the chemotherapeutic agents efficacy and mechanism of action [87].

### 2.3. Vibrational Spectroscopy as A Diagnostic Tool

#### 2.3.1. Diabetes and Obesity

Diabetes denotes a pathologic condition depicted by elevated blood sugar concentrations over a sustained period of time. This malady is due to either insufficient insulin production by the pancreas or by failure of the body cells to respond normally to the produced insulin (type 1 or type 2 diabetes, respectively). The severe long-term faced complications, including stroke, cardiovascular disease, foot ulcers, chronic kidney disease, and damage to the eyes, could be avoided if accessible and effective methods to detect diabetes or pre-diabetic stage, potentially reversible, would be available.

Several reports have shown that branched-chain aminoacids (BCAA), involving leucine, isoleucine, and valine have elevated levels in insulin-resistant and type 2 diabetic and obese patients body fluids (plasma and urine), therefore making them promising candidates as risk predicting biomarkers for type 2 diabetes [104,105,106], in the first four years. Birech et al. [107] have explored by Raman spectroscopy the leucine and isoleucine amino acids potential as diabetes type 2 biomarkers. Furthermore, they have evaluated the Raman spectroscopy ability to validate the anti-diabetic drug efficacy. Prominent Raman peaks associated with glucose, leucine, and isoleucine amino acids were present in blood as also synthesized by Table 2.

The Raman bands at 1125, 1395, and 1437 cm^−1^ displayed decreased intensities in blood after the administration of anti-diabetic drugs to diabetic rats, indicating the reduction in concentration levels of the corresponding biomarker molecules glucose, leucine, and isoleucine, as reported by the authors [107]. The results of this study allowed the claim that Raman spectroscopy has a significant potential in diabetes screening, based on the blood levels of leucine and isoleucine. It was also revealed that, using Raman spectroscopic signatures of the aforementioned amino acids, the method can be a favorable alternative to assess the efficacy of new anti-diabetic drugs through comparative studies.

Obesity, strongly correlated to diabetes condition and many other diseases, contributes to functional infirmities in liver or muscle tissues. As already established in earlier sections, disease conditions produce variations of structure, composition, concentration, and function of specific biomolecules directly revealed in the vibrational spectrum and can be evaluated using vibrational spectroscopy practices [114]. This hypothesis is applied also for obesity disorder, since IR identifies the molecular modifications in order to understand the disease development and to detect specific diagnostic spectral biomarkers.

The impact of adipose tissue investigations in obesity and obesity related disorders diagnosis and therapy by IR was highlighted recently. Furthermore, the results of some studies on adipose tissue samples will be brought into question in order to highlight the spectral changes induced by obesity. A typical human adipose tissue IR spectrum includes different functional groups related to biomolecules of the sample, with the fingerprint region established in the 1800 − 750 cm^−1^ region [86]. The relevant assignments of the peaks found in the spectrum of human adipose tissue are assumed in Table 3 [115,116].

The appearance of CH_2_ stretching bands offers information about lipid flexibility, while the bands position shift provides structural information. In this regard, the CH_2_ bands shift, either symmetric or asymmetric, refers to the lipid acyl chains variability and may indicate lipid disorders [117,118]. The C=O and asymmetric PO_2_^−^ stretching shifts are related to their hydration level [118]. The amide I position shift along with the amide I/amide II ratio are in a strong connection with modifications in protein secondary structure [119].

#### 2.3.2. Cancer

Vibrational spectroscopic techniques have extensively contributed a significant number of research works concerning the diagnosis and screening of cancers but with negligible application in clinics nevertheless.

(1) Cervical Cancer

Since cervical cancer is positioned as the 4th most common form of cancer in women worldwide with an estimated of 570,000 new cases in 2018 and about 310,000 deaths [120], the early stage detection of development and pre-malignant state still remains a medical priority at the present time.

ATR-FTIR, a well-known method to analyse the chemical structure and interfaces of proteins, lipids, nucleic acid, and carbohydrates biomolecules, proved to be a powerful alternative for spotting spectral variations in pre-malignant and malignant cells [121,122,123], allowing scientists to explore the potential of new biomarkers or spectral fingerprints. Neves et al. [124] have used ATR-FTIR for differentiation from blood plasma of NILM (negative for intraepithelial lesion or malignancy) and SIL (squamous intraepithelial lesion) and to divide the cervical squamous intraepithelial lesions into low-grade and high-grade (LSIL and HSIL). In the area between 1800 and 900 cm^−1^, called the “bio-fingerprint region,” the major typical IR absorption bands were found as follows: the 1650 cm^−1^ and 1550 cm^−1^ peaks were allocated to amide I and amide II from amino acids and proteins; the 1400–1470 cm^−1^ range was dedicated to the methylene groups of lipids; 1225 cm^−1^ and 1080 cm^−1^ are symmetric and asymmetric phosphate stretching vibrations; the less intense peaks at about 1155 cm^−1^ were related to C−OH and C−O moieties existent in serine, tyrosine, and threonine amino acids and carbohydrates; and around 1030 cm^−1^, a smooth band can be observed, assigned to glycogen [125]. The initial obtained spectra were preprocessed by normalization and baseline correction features, followed by the classification models (PCA-LDA/QDA, SPA-LDA/QDA, and GA-LDA/QDA) that were assembled on both the raw and processed data in order to compare the results. Using the GA-LDA/QDA model, the datasets classified NILM vs. SIL, NILM vs. LSIL, and NILM vs. HSIL as presented in Table 4, with good values of sensitivity and specificity (80%–100%) [124].

FTIR spectroscopy has been also applied directly to cervical cells to confirm the malignant fingerprints. Usually, the transmission measurement is recorded on tissues or/and cells that are placed on infrared transparent non-hygroscopic crystal windows based on calcium fluoride or barium fluoride. The FTIR spectrum was obtained, comprising the biomolecules of a typical ectocervical cell (protein, lipid, glycogen, DNA, and RNA), besides the separate spectra of these macromolecules. The major bands assignments were also provided in ectocervical cell-labelled spectrum that are summarized in Table 5.

The analysed spectra exhibited well-defined modifications in the glycogen and phosphodiester region (1300 − 950 cm^−1^) among dysplastic or malignant and normal cells, as many research works have revealed to date [126,127]. Morris et al. [128] revealed that, in CIN III samples (with high risk of developing carcinoma), an extra peak at 972 cm^−1^ was detected, suggesting it to be a key malignancy marker. On the other hand, this band can be detected in any cell type with a high amount of nucleic acids; thus, this piece alone cannot be considered a spectral indicator for CIN III. Wood et al. [129] joined FTIR with PCA to discriminate normal from dysplastic cervical cells. The essential problem regarding the correlation between cytological diagnosis and the FTIR method represents the large number of false-positive and false-negative fallouts related to the Pap smear [130].

Currently, data processing of a spectrum eliminates the spectral segments materialised due to physical outcomes from the chemical constituent. In this respect, using the RMie-EMSC algorithm (Resonant Mie Scattering-Extended Multiplicative Signal Correction), the wavelength-related refractive index changes and the particle size are modelled [135,136].

In the case of such approaches, it could be concluded that FTIR spectroscopy, although providing important information about cell composition, presents certain limitations so it needs to be supplemented with other investigation techniques, from FTIR imaging and screening to pathologists and subsidiary diagnostics such as flow cytometry, immunohistochemistry, or polymerase chain reaction. While the “gold standard” is still lacking in reliability, the infrared spectroscopy, with the advantage to involve small tissue or cell sample of minimal preparation, may be considered valuable as a prognostic indicator of cervical cancer.

(2) Oral Cancer

Oral malignancies are among the 6th most common cancers with an increasing incidence rate. A number of new cases (275,000) are recorded yearly, mainly in developing countries, due to the population habits to use carcinogens, such as tobacco and betel quid. Oral squamous cell carcinoma (OSCC), identified in all oral cancer cases, develops from visible oral mucous membrane lesions like leukoplakia (OLK) and erythroplakia [137,138,139,140,141,142,143,144,145,146,147,148,149]. Other forms of oral precancer or premalignant lesions are oral submucous fibrosis (OSF), tobacco pouch keratosis, or lichen planus (OLP) [140].

Bakker Schut et al. [141] used Raman spectroscopy for the first time in oral cancer investigations since the technique is sensitive to molecular composition and conformation changes that arise in tissue during carcinogenesis. The authors induced in vivo on a rat model dysplasia in the palate epithelium using 4-nitroquinoline 1-oxide topical applications in order to achieve a classification of normal and dysplastic tissue. The results of the study were encouraging, presenting for the first time a method with high sensitivity and specificity able to detect high-grade dysplasia in vivo within the 10th measurement.

Venkatakrishna et al. [142] performed the next study that used Raman spectroscopy in oral cancer diagnosis. This time, the authors analysed human samples, more precisely, oral frozen cancer biopsies; the results highlighted that the spectra of normal tissue samples look like those of lipids (C–C, C=C, and C=O), while the malignant tissue samples are similar with protein spectra (amide I, amide II, and phenylalanine). Since other ex vivo studies on both fixed and frozen tissues had positive results, in vivo oral cancer studies on humans were initiated also by Guze et al. [143] with the aim to identify deviations position in the oral cavity [140].

FTIR has proved to be a significant tool for carcinoma detection through chemical variations examination at molecular level [144].

Naurecka et al. [145] compared in 2017 the FTIR-ATR and FT-Raman absorption spectra of abnormal (OLK and oral cancer) and normal tissues in the main regions of lipid (2800–3000 cm^−1^), protein (1500–1700 cm^−1^), and nucleic acids (1000–1250 cm^−1^), pointing out the OLK “fingerprint region” is in the range of 900–1800 cm^−1^. The FTIR spectrum revealed the following modifications: the absorption peak at 1238 cm^−1^ is correlated with nucleic acids symmetrical stretching, the phosphate wavenumbers were notable lower compared with that of normal tissue, and the shifting of the 1030 cm^−1^ band was ascribed to –CH_2_OH vibrations (1024 cm^−1^ for OLK and 1030 cm^−1^ for cancer/normal tissue). In the Raman spectrum, additional absorption peaks at 1320 cm^−1^ (amide III of protein or collagen) and 1248 cm^−1^ (PO_2_^−^ asymmetric vibrations) have been identified. The obtained data indicated that both FTIR and FT-Raman methods have real potential to be applied in the clinical diagnostics of oral cancer and as basic examination at the molecular level. Quick detection and screening for OLK reduces mortality; therefore, these two complementary techniques could give an easy, fast, and accurate diagnosis. Further studies using both FTIR and Raman must be undertaken to ensure efficiency and repeatability of the methods.

Rai et al. [146] investigated using FTIR analysis another pre-malignant oral lesion OSF. The study involved 60 subjects divided into two groups: OSF patients confirmed by histopathological methods and apparently healthy patients, as controls. Remarkable FTIR spectra differences between the two groups have been identified in 45 infrared wavenumbers, describing variations in proteins, lipids, carbohydrates, and nucleic acids. The peaks identified at 1045 and 1025 cm^−1^ that correspond to the –CH_2_OH groups and to the C−O stretching and bending of the C–OH moiety of carbohydrates indicate a rich glycogen superficial layer. The changes in amide FTIR spectra regions amide I/collagen (1650 and 1035 cm^−1^), amide II (1544 cm^−1^), and amide III (1313 cm^−1^) may be correlated to the atypical amino acids and albumin concentration in serum of OSF patients.

(3) Gastrointestinal Cancers

Gastrointestinal (GI) cancer, a globally common condition, is a term that refers to 10 types of cancers found in the digestive system: esophageal cancer, gastric cancer, pancreatic cancer, neuroendocrine tumours, small bowel cancer, liver cancer, gallbladder and biliary tract cancer, GI stromal tumour, colon/colorectal cancer, and anal cancer. In USA, GI cancers are the second leading cancer death cause, after lung cancer [147].

● Colon/Colorectal cancer

Colon cancer, and more generally colorectal cancer (CRC), is a key clinical problem positioned in the 3rd place of cancer death worldwide, according to the American Cancer Society. Annually, 1.5 million new cases are discovered, half of them dying in a few years [148]. Colonoscopy is the most widely used technique for monitoring CRC [149,150].

Li et al. [144] have analysed in vivo and in situ normal and malignant CRC tissues using FTIR spectroscopy. The normal tissue spectra are interpreted as follows: symmetric and asymmetric stretching vibrations of –CH_2_ near 2852 cm^−1^ and 2930 cm^−1^, –CH_3_ at 2873 cm^−1^ and 2958 cm^−1^, and C=O stretching vibration at 1745 cm^−1^. In the spectra of malignant tissues, the abovementioned peaks usually decrease or vanish, due to the fact that triglyceride contains a significant proportion of the three mentioned groups that is consumed by the carcinoma increased nutritional and energy requirement.

Dong et al. [151] have also proved that the FTIR-ATR technique can be a practical tool to discriminate malignant and normal CRC tissues. The study displayed increased peak intensity for cancer compared with normal tissue and higher wavenumbers peak positions for malignant tissues. The authors highlighted an increased value for malignant tissue compared to normal ones at the 3260 cm^−1^ vibrational band, related to protein OH and N–H stretching vibrations, explained by the fact that malignant tissues contain accumulate important amounts of water and mucus, as can be seen in Table 6. In the 1700 − 1500 cm^−1^ region, linked to amide I and II bands of proteins, the authors noticed important variation between the normal and malignant colon tissue. The 1640 and 1550 cm^−1^ vibrational bands from N−H bending were attributed to the fact that colon adenocarcinoma secrete excessive mucus. The band at 1080 cm^−1^ (related to PO_2_ group) increased significantly in the malignant group, mainly due to the fact that, in cancer cells of malignant tissues, DNA replication is endless.

● Gastric cancer

Gastric cancer (GC) is the fifth in terms of incidence rates and the third in terms of mortality, compared to other severe malignant tumors. Even if incidence rates and mortality of the gastric cancer are constantly decreasing, this malignancy still has a remarkable threat to human health due to a poor diagnosis and prognosis for GC patients [154].

Gastric tissue specimens from endoscopic biopsies (chronic superficial gastritis, chronic atrophic gastritis, cancer, and healthy stomach tissue) have been investigated using FTIR-ATR. The spectral features were identified in the 1800 − 1000 cm^−1^ region. The shape of the chronic superficial gastritis related spectrum was similar to the one for healthy gastric tissue. The notable band at 1640 cm^−1^ is correlated to the protein amide I and to the H−O−H bending vibration of water, while the band at 1550 cm^−1^ is associated to N−H bending and C−N stretching of amide II proteins [152]. Amide I and II bands intensity are higher in the healthy gastric tissue spectrum than in the chronic superficial gastritis tissue because healthy gastric tissue contains regular protein secondary structures, like the α-helical structure. The intensity of the absorption peak at 1310 cm^−1^ increased in the cancer spectra. The spectra of atrophic gastritis samples showed a weaker absorption peak nearby 1310 cm^−1^, correlated with the malignant tissues spectra [153].

● Liver cancer

Worldwide, liver cancer occupies the third cause of mortality from cancer, mainly due to the identification in advanced stage. The clinical practice depicted the most common risk factors associated with liver cancer initiation, development, infection of hepatitis B virus (HBV) and hepatitis C virus (HCV), high fat diet, heavy alcohol consumption, and aflatoxin B1 exposure. Among all types of liver cancer, hepatocellular carcinoma (HCC) represents the major type of primary liver tumour with an increasing incidence, followed by cholangiocarcinoma (CCA) as the second one, comprising 10% of liver cancers with a reduced survival rate [155].

Using SERS, 56 human liver tissue slices were analysed in the 500–1800 cm^−^^1^ fingerprint region. Important changes in the absorbance intensities of the cancerous tissues have been observed for many characteristic vibration peaks. The biomolecule contents corresponding to 1663 cm^−1^ (C=N stretching for DNA, protein, and collagen I), 1634 cm^−1^ (C=O stretching of amide I), 1485 cm^−1^ (ring breathing modes; major assignment: DNA, adenine, and guanine), 1336 cm^−1^ (CH_3_CH_2_ twisting of DNA, collagen), and 928 cm^−1^ (C−C stretching of protein, proline, and valine) were higher in liver cancer tissues when compared with normal tissues. On the other hand, the diagnostic signals equivalent to 1585 cm^−1^ (C=C stretching), 1448 cm^−1^ (CH_2_CH_3_/CH_2_ deformation), 1158 cm^−1^ (C−C/C−N stretching), 907 cm^−1^ (COC stretching of formalin), and 838 cm^−1^ (CC skeleton telescopic of amine groups) were lower in cancerous liver tissues than in normal tissues. Also, the specific vibration peaks at 1022 and 1048 cm^−1^ related to glycogen are more intense in the cancerous tissue. Other important issues observed in SERS spectra were the significantly altered differences among the liver cancerous and normal groups correlated to the content of DNA (1663, 1485, 1373, and 1336 cm^−1^), suggesting a DNA or RNA bases atypical metabolism [156].

(4) Bone Cancer

Primary bone tumours, such as plasmacytoma are identified in clinical practice more rarely than metastases from carcinomas, melanoma, or hematologic malignancies. The non-neoplastic bone conditions, like inflammatory processes, non-ossifying fibroma, fibrous dysplasia, bone cysts, and Paget’s disease, surpass the primary bone tumours. The majority of malignant bone tumours are formed based on a hereditary disorder but can also occur spontaneously with a recurrence degree classified as follows: 35% for osteosarcoma, 25% for chondrosarcoma, 16% for Ewing’s sarcoma, 8% for chordoma, and 5% for malignant fibrous histiocytoma [157].

A total of 20 bone specimens obtained from 10 Ewing’s sarcoma patients were examined by Chaber et al. [158]. Half of the samples were collected before the treatment considered diagnostic biopsy sample and half were from the resected tumour after neo-adjuvant chemotherapy; all the samples were paraffinized. The obtained spectra from paraffin and deparaffinized bone samples did not reveal any significant issues. The comparison of the bone spectra before and after chemotherapy identified variations in the peak absorbance values and also in spectrum shape. Specific peaks have been observed: 3283 cm^−1^ peak related to NH and OH stretching of the proteins peptide bond (–NHCO–) and functional groups of water; 1635 cm^−1^ amide I attributed to coupled C=O stretching and N–H bending of proteins; 1540 cm^−1^ for amide II (proteins N–H bending and C–N stretching); 1396 cm^−1^ correlated to carbonate υ_2_(CO_3_^2−^); 1234 cm^−1^ for amide III (proteins δ N–H bending, C–N, and C–C stretching); and 1162 cm^−1^ related to proteins C–O stretching form of threonine, serine, and tyrosine of protein EE [158].

(5) Breast

Breast cancer remains the second death source by cancer in females, after lung malignancies, with numerous new yearly cases [159]. For most of the cancer patients, long-lasting survival matches with early diagnosis, and for this reason, new, noninvasive methods of detection are needed. Raman spectroscopy, based on previous century’s theory and due to last decades’ research and technology evolution, started to give promising results for early diagnosis and treatment [160].

In 2013, Liu et al. [159] used NIR Raman spectroscopy for detecting the modifications of molecular composition given in malignant breast tissue. The authors highlighted the beneficial effect of the resonance Raman spectroscopy that occurs when the energy of an approaching photon rises adjacent to the electronic transition energy of the electrons linked with the corresponding molecule bonds. In the situation when the laser beam frequency overruns the electronic transition energy degree, the molecule’s transition vibrational modes are highly amplified and the resulting spectra display intensification of the Raman scattering. The given example was flavin molecules that are excited with 532 nm of light and give a large vibrational mode associated with their bonds, while others remain unaffected. Those findings are useful for the detection of big molecules with fewer peaks but of superior resolution. Resonance Raman spectroscopy can efficiently detect the spectral features in biological tissues, the vibrations with weak signals, and biological changes on molecular level and can target vibrational groups (collagen, elastin, tryptophan, and NADH) in cells. The examined samples were normal breast, benign tumour, and ductal carcinoma tissues. The separation among normal and cancer breast cells were identified in the peak positions as well as at the intensity ratios of the 700–1800 cm^−1^ Raman spectral area. The values of the distinct peaks for normal breast tissue were 1156, 1521, 2854, and 3013 cm^−1^. The amide II peak at 1548 cm^−1^ was more intense for the cancer tissue, among other 12 peaks in the region 500–1800 cm^−1^. The statistical multivariate analysis and the high level of specificity and sensitivity in tissue differentiation established in this study the clinical value of Raman-based technologies [160].

In the same year, Surmacki et al. [161] published their study on breast cancer diagnosis using confocal Raman and infrared spectroscopy. Dissimilarities between normal breast and malignant tissue were identified in typical regions of proteins, fatty acids, carotenoids, and interfacial water vibrations. The spectra of Raman for non-malignant tissue showed peaks at 1158 and 1518 (carotenoids); 1444, 1660, 1750, 2854, 2888, 2926, and 3009 (fatty acids); and 877, 1004, and 1304 cm^−1^. Cancer tissue peaks were identified at 558, 1098, 1269, 1444, 1660, 2888, 2926, 2940, and 3311 cm^−1^. The study depicted that cancerous tissue has predominant protein component [161].

Focal-plane-array (FPA)-FTIR microspectroscopic technique combined with PCA was used as a diagnostic approach for monitoring chemotherapy effects in triple-negative breast cancer patients [162]. The results revealed that the primary bands accountable for the breast cancer tissue sections discrimination pattern before vs. after chemotherapy were those related to amide I and II bands, namely 1654 cm^−1^ (α-helix), 1462 cm^−1^ ((CH_2_) from methylene (–CH_2_) groups), 1411 cm^−^^1^ ((CH_2_) of disubstituted cis-olefins), and 1049 cm^−^^1^ (ν(C–O) coupled with δ(C–O) of C–OH groups of carbohydrates mainly from glycogen) evidencing that proteins are most susceptible to mutation during carcinogenesis. It is important to mention that the FPA detector is considered to herald a new era for FTIR utilization in the biosciences [163].

Nicolson et al. [160] published last year their research on “surface enhanced spatially offset resonance Raman spectroscopy” (SESORS), combining two Raman technologies. More specifically, SERS and SORS put into use a reporter with an electronic transition with the laser frequency in resonance, resulting in more powerful Raman signal. Their experimental study established better quality of imaging through boosted Raman signals in deeper tissue levels, being able to detect tumours on a breast cancer porcine tissue model. Moreover, they used multicellular tumour spheroid nanotags for a more precise localization of the disease.

As the technology evolves, these findings could move forward to human applications in the near future.

(6) Skin

The vast number of new skin cancer cases in recent years determined the researchers to find more accessible procedures for an early detection in skin tissue, to predict the evolution of pre-cancerous lesions, to identify the margins of tumor lesions in real time during surgical therapy, to rationally select a suitable molecular therapy, and to monitor the response to therapy at a molecular level [164,165].

Peñaranda et al. [166] have guided an extensive analysis aiming to emphasize the capabilities of FTIR spectroscopy to differentiate normal from malignant skin cells. This research has involved several cell lines, two normal (HaCaT-keratinocytes, Homo sapiens and NIH-3T3 Embryo Fibroblasts, Mus musculus) and two skin malignant melanoma (A-375 and SK-MEL-28, Homo sapiens). The preprocessing was normalized and standardized in order to characterize the samples from a biochemical point of view and to neglect the physical assets such as concentration, thickness, and morphology. One of the main distortions was found in the amide I (~1600–1700 cm^−1^)/amide II (~1500–1600 cm^−1^) ratio or derivative-like depressions outside the amide I peak.

Previous studies assessed that cell FTIR spectra can be differentiated according to their cell cycle stage, including the apoptotic cells that could be present in cell culture due to the stress it was exposed to [167,168].

According to the obtained data in the field, further advance of standardization protocols is needed in the effort to integrate and adapt the current procedures in clinical laboratories. Consequently, the development of a reliable diagnostic result should use FTIR fingerprint from cells and tissues with a reference delivered by pathologists, similarly to histopathology assays.

Many other studies, which are based on the same concept that any tumour lesion is invariably accompanied by biochemical changes at the cellular level, have paid special attention to vibrational spectroscopy as an alternate, easier method of investigation. Table 7 comprises the main structural changes observed in IR and Raman spectra of different skin tumour cells: melanocytic lesions (MM), SCC (squamous cell carcinoma; SCC in situ and invasive SCC) and BCC (basal cell carcinoma), pigmented nevi (PN), and seborrhoeic keratosis (SK).

As Table 7 depicts, data obtained using NIR-FT Raman spectroscopy showed noticeable differences between normal samples and samples of malignant, premalignant, and benign skin wounds. Most of the skin lesions have exhibited alterations in the 1065–1094 cm^−1^ and 1243–1258 cm^−1^ spectral regions that were assigned to phosphate backbones, phospholipids, and reflect protein conformations [174]. The alterations of protein structures are not indications of malignancy as NIR-FT Raman spectroscopy suggests but the alterations of α-helix structure expressed by a global decrease in total intensity for the amide I, II, and III regions in the BCC probes were described [173]. The spectra of MM were differentiated from PN, BCC, SK, and control skin essentially for the amide I protein band at about 1660 cm^−^^1^ decreased intensity [173].

Lately, multivariate data assessment methodologies like PCA, factor analysis (FA), soft independent modelling of class analogies (SIMCA), or artificial neural networks (ANN) analysis were enforced to identify typical spectral signatures but also to expand the reliability of diagnosis. In this respect, Gniadecka et al. [173] reported that, using ANN of Raman spectra, remarkable diagnostic accuracy parameters were achieved, namely sensitivity of 85% and specificity of 99% for MM and of 97% and 98% for BCC, respectively; SK and PN were diagnosed with 96 and 78% sensitivity, respectively.

According to these studies, we can conclude that IR and Raman spectra obtained from benign, premalignant, and malignant skin lesions display analogous alterations in the regions responsible for lipids, proteins, and nucleic acids. As a consequence, it is very feasible that malignant transformations have generated similar molecular changes in the tissue involved. Furthermore, by applying different multivariate statistical models and by using customized software packages, the presence of a skin neoplasm could be detected along with the possibility to discriminate distinctive types of skin tumours [175].

Overall, IR techniques could be used in cancer diagnosis, since normal/malignant tissues are analysed through the spectra of their complex composition and the tissues do not need pre-treatment or labelling and are characterized in real-time. Particular differences have been found between IR modalities, briefly detailed as follows:

- increased sensitivity of Raman to homo-nuclear functional moieties;
- increased sensitivity of FTIR to hetero-nuclear molecular groups and polar chains.

Compared with normal tissues, in the spectra of malignant tissues, several peaks can decrease/increase or even disappear due to specific modifications usually found in the chemical composition of malignant tissues: abnormal concentration of amino acids are found in malignant tissues; malignant tissues contain increased amounts of water and mucus compared with normal ones; in cancer cells of malignant tissues, DNA replication is endless; and in the area of malignant tissues, increased nutritional and energy requirement are identified.

#### 2.3.3. Neurological Disorders

Neurological disorders denoted by Alzheimer’s and Parkinson’s disease, dementia, epilepsy, multiple sclerosis, neuroinfections, headache disorders, stroke, traumatic brain injuries, as well as brain tumours constitute a large and increasing public health problem with increased burden to the patients, families, and also society. In 2016, neurological disorders were the second leading cause of deaths globally, with a high percentage of 16.5% deaths from all causes [176]. According to World Health Organization (WHO, 2016), the prevalence of neurological disorders is expected to increase in the near future owing to population growth and aging, placing pressure on already overloaded health-care services and resources [177]. Therefore, there is an urgent need to develop new strategies to improve prevention and management of degenerative diseases across the globe.

Reviewing the potential of vibrational spectroscopy, a significant number of scientific papers explore the neurological disorders namely Alzheimer’s and Parkinson’s disease, epilepsy, multiple sclerosis, bipolar disorder, schizophrenia, cerebral malaria, ischemic stroke, hemorrhagic stroke, and depression in human and animal models. The published abundant literature data demonstrates the correlation of biochemical changes, such as protein misfolding and aggregation, lipid oxidation, abnormal carbohydrate metabolism, and DNA/RNA unusual expression, monitored by vibrational spectroscopy techniques with neurological diseases.

Modern infrared methods have been applied in neuroscience since 1993 by Wetzel and Le Vine [178], which examined brain tissue frozen sections from mice using in situ FTIR-MSP. The studies revealed significant chemical differences between the white and gray matter IR spectra of normal brain specimens established on the presence or absence of myelin. In healthy tissue, the white matter includes high myelin levels compared to the grey matter. The spectra obtained by spatially resolved in situ probing of cerebrum section indicated that the white matter could be differentiated from the grey matter by specific elevated absorbance infrared bands at 2927, 1740, 1469, 1235, and 1085 cm^−1^, respectively (Table 8). Both materials showed similar absorbance at 1550 cm^−1^ related to amide II. In Krabbe’s disease, FTIR-MSP was employed to localise psychosine in the twitcher mice brains and to link chemical changes to histopathology ones. The broadening of the CH_2_ band to 2919 cm^−l^ in only twitcher mice brains and not in normal mice compared with the absorbing psychosine band at 2919.6 cm^−1^ have established the accumulation of psychosine. This finding is in good agreement with the biochemical analysis that detected higher psychosine concentrations in the hindbrains compared to the cerebra [179].

It is well known that Alzheimer’s disease is measured by amyloid beta (Aβ) peptide accumulation with characteristic β-pleated sheet fibril structure in brain grey matter areas. Traditionally, Alzheimer’s disease clinical diagnosis on patients is built upon neurophysiological observations, positive positron emission tomography (PET) amyloid imaging, neuro-imaging computerized tomography (CT) and magnetic resonance imaging (MRI) techniques, low cerebrospinal fluid (CSF) Aβ_42_, diminished ^18^FDG (flurodeoxyglucose) uptake in specific regions of the brain, elevated CSF tau, and rarely histopathological assessment [180] of either biopsy or postmortem. At this time, no reliable laboratory investigations can be performed to differentiate the pathology; therefore, new methods are tested to make a certain diagnostic.

The first proof of the β-amyloid proteins in situ existence within a slice of diseased Alzheimer’s human brain tissue was done by Choo et al. [181] using the synchrotron FTIR-MSP technique. The spectroscopic mapping of brain tissue areas showed that in situ protein of grey matter exists in a β-amyloid structure but also in α-helical and/or unordered conformation, differentiated by IR peaks (Table 8). The use of synchrotron FTIR-MSP offered the possibility to observe the subtle differences in amide I band and to enhance spatial resolution without fading the signal-to-noise ratio when compared with conventional IR-MSP. Standard histological techniques using Congo red dye that exclusively marks amyloid in reddish-pink were performed to confirm the β-amyloid extracellular deposit.

The synchrotron-based IR-MSP was used by Miller et al. [182] to show the focalized deposition of Cu and Zn metal ions with β-amyloids in Alzheimer’s disease correlated with protein misfolding in two neuropathologic autopsies. The FTIR spectrum from an Alzheimer’s disease plaque shows regions (amide I at 1625 cm^−1^) of high β-sheets in contrast with the unaffected area; the results are confirmed by the Thioflavin dye binding to amyloid fibrils. Elevated levels of the metal ions found within the brain using X-ray fluorescence microprobe sustained their association with Alzheimer’s disease hypothesis. The authors recommend that further studies must be made for evaluating the cascade of actions that lead to the co-localization of β-protein and Cu and Zn ions in amyloid plaques.

In 2006, Miklossy et al. [183] exposed in vitro neuronal cells and mammalian glial from Sprague–Dawley rats to *Borrelia burgdorferi* spirochetes and to the inflammatory bacterial lipopolysaccharide to determine the pathological mechanism driving the accumulation of β-amyloid in Alzheimer’s brain. Synchrotron IR-MSP assessed the presence of β-amyloid conformation in the *Borrelia*-induced amyloid deposits; the results were confirmed by histochemical and immunohistochemical analysis. The intensification of the 1630 cm^−1^ near region of β-amyloid analysed following 4-week exposure to spirochetes absent in the control samples corresponds to thioflavin S-positive plaque in the cell cultures. The study indicates that bacteria presence boosts key-chain actions heading to amyloid accumulation in Alzheimer’s disease.

The same techniques, synchrotron FTIR-MSP, examined the cortex, hippocampus, and caudate tissue of TgCRND8 amyloid precursor protein transgenic mice [184]. The authors compared in situ diffuse and dense-core Aβ plaques and correlated the protein secondary assembly and chemical composition with tissue morphology in order to provide new insights into the disease process. Spectral analysis of dense-core plaques displayed increased intensity in the amide I area at 1623 cm^−1^ corresponding to highly aggregated β-sheet content. It is essential to mention that the amide I found at TgCRND8 mouse plaque cores fluctuates from a maximum of 1632–1634 cm^−1^ related for human Alzheimer’s disease plaques [181] due to biochemical or structural dissimilarities but is similar to in vitro Aβ fibrils. The authors also evidenced that dense-core plaques are bordered by phospholipids in considerably increased amounts, probably as an outcome of cellular feedback to aggregated amyloid. The results were sustained by histochemistry and immunostaining analysis that revealed and visualized the occurrence of vascular amyloid accumulations in TgCRND8 mice.

Parkinson’s disease is a chronic gradual disease described by dopaminergic neurons loss and the Lewy bodies’ presence in the brain substantia nigra. Currently, Parkinson’s disease clinical diagnosis depends on the observation of the distinctive motor symptoms, bradykinesia, tremor, and limb rigidity with only postmortem examination [185]. High lateral resolution synchrotron radiation based-FTIR-MSP was also employed for biomolecular evaluation of human substantia nigra in normal and Parkinson’s disease tissues [186]. The infrared spectrum of control tissue shows that the proteins and nucleic acids functional groups (Table 8) are primarily detected in the body cell, while the phospholipids band at 1740 cm^−1^ was placed in the nerve cell body exterior, along with the lipids functional moeties at 2930 and 2850 cm^−1^ outside the neuron area. The FTIR absorption spectra typical obtained for Parkinson’s disease shows no deviations between cell body and the surroundings of any of the bands. Particularly, the functional groups that display fluctuations in Parkinson’s disease in comparison with the control are as follows: higher intensity of the lipids at 2930 and 2850 cm^−1^ and of the band at 1173 cm^−1^, shifted amide I region (1643 and 1682 cm^−1^), marked differences in the 1000–1380 cm^−1^ area (amide III, nucleic acid, and carbohydrates), decrease of protein to lipid ratio, and also intensification of amide I/amide II ratio.

New perceptions into the multiple sclerosis neuropathology were gained using FTIR-MSP, bioinformatics, and a synchrotron light source by analysing fluctuations at macromolecular level in the central nervous system through the induction and prevention of autoimmune-mediated demyelination on animal model [187]. Multiple sclerosis is an autoimmune neurodegenerative disease that disturbs the myelin sheath, triggering localized inflammation, followed by demyelination, and finally axonal injury of the central nervous system [188]. FTIR images of autoimmune encephalomyelitis lesions exposed low lipid concentration and high nucleic acid and displayed modifications in their protein secondary structure (Table 8) when compared with control animals. The results illustrate the potential of FTIR as a supplementary, complementary “gadget” that can be directly linked with standard procedures such as immunochemistry in the examination of multiple sclerosis pathology.

A more recent study developed an innovative approach for differential diagnosis of relapsing-remitting multiple sclerosis (RRMS), clinically isolated syndrome (CIS), and CIS to RRMS transformed (TCIS) patients by IR connected with multivariate analysis from cerebrospinal fluid [189]. Spectral, HCA, and PCA evidenced the biomolecular differences such as significant intensification in carbonyl group and reduction of amide I/amide II and lipid/protein ratios and the appearance of a new band placed at 795 cm^−1^ fitted to guanine C3′-endo/syn conformation in the Z-DNA only in the diseased CSF groups. Therefore, the results suggest that FTIR spectra statistical and computational analyses could be used as complementary tools for the early diagnosis of RRMS patients.

In 2012, Caine et al. [19] quantified the absorption band positions of the main functional group identified in brain tissues, briefly detailed below:

- the bands in the 3050 − 2800 cm^−1^ region are subject to antisymmetric and symmetric CH stretches of methyl, methylene, and methine moieties from lipid and proteins;
- the absorption bands starting from 1700 to 1500 cm^−1^ region are dedicated to proteins;
- the absorption bands identified in the 1350 − 1000 cm^−1^ area were ascribed to phosphate groups and carbohydrates.

Based on the distribution of typical absorption bands acknowledged in brain tissues, the review paper highlights the ability of FTIR-MSP combined with bioinformatics to identify protein secondary structural variations linked with neurons in Parkinson’s disease, Alzheimer’s plaques, and meningioma on clinical models, as well as in Alzheimer’s disease animal models, multiple sclerosis, and transmissible spongiform encephalopathies.

In 2014, Surowka et al. [190] studied the protein and lipid composition unpredictability of human subtantia nigra in aging by means of FTIR-MSP. The evaluation was carried out on 35 autopsy human samples without any signs of neurologic disorders. The results showed that the total content of proteins and lipids decreases with age of individuals. Significant differences were also observed between the anti-parallel β-sheets content in the neuron bodies and extra neuronal spaces from 7th to 8th life decade. In this context, the authors underline the “biochemical link” between the etiology of the Alzheimer and Parkinson’s diseases and physiological aging through several pathological lipid and protein-linked processes.

Ogruc Ildiz et al. [199] proposed FTIR coupled with multivariate analysis as a supplementary diagnostic tool for of schizophrenia and bipolar events in 2016. Briefly, bipolar disorder is described by abnormal shifts in energy, mood, and activity levels and schizophrenia is described by hallucinations and delusions, amotivation, or negative symptoms with clinical diagnosis based only on psychopathology. IR spectra of 30 blood plasma aliquots from bipolar, schizophrenic, and control patients were collected and analysed, and the spectral differences as depicted from PCA were correlated with disease biomarkers. Three types of spectra were identified and detected mainly in the 3750 − 2800 cm^−1^ and 1300v950 cm^−1^ region and described specifically as follows: lipids at 2850 and 2920 cm^−1^ assigned to C–H stretching of CH_2_ and CH_3_ groups, the peaks at 1080 and 1242 cm^−1^ attributed to the symmetric and asymmetric phosphate groups (P–O) from DNA component, and the band at 3290 cm^−1^ ascribed to NH stretching vibrations. Partial least square-discriminant analysis (PLS-DA) model classified and differentiated the blood samples using whole spectral range and regions I and II with high sensitivity and specificity values. The authors suggest that this methodology should be applied as an assisting tool for the identification and refinement of mental diseases.

Many authors have investigated vibrational spectroscopy techniques in clinical studies as a diagnostic method for cancer to monitor the treatment progress or postoperative outcomes for patients [19,200].

Since the identification of tumor margins minimizes the potential for recurrence, additional control with a spectroscopic probe to detect real time in situ invasive brain cancer is mandatory. Jerym et al. [201] detected intraoperative brain cancer using handheld fiber optic Raman spectroscopy probe on 17 patients with glioma by accurately differentiating with 93% sensitivity and 91% specificity healthy brain from condensed cancer and normal brain attacked by cancer cells. The imaging findings were compared with the obtained biopsy specimens, making the Raman spectroscopy probe an ideal tool for decision-making in surgical resection. Lakomkin and Hadjipanayis [202] published in May 2019 a useful review on the handheld spectroscopic tools used in neuro-oncologic surgery. Succinctly, the authors present the handheld Raman spectroscopic tools in discriminating tumour from healthy brain tissue, as a complementary tool to intraoperative resection on animal models and clinical studies. The features of the elected studies on the role of Raman in identifying different types of brain tumors in the operative setting reported sensitivity and specificity above 90%, highlighting the accuracy in both human and animal specimens.

Recently, Lin et al. [203] published a state-of-the-art analysis that employs FTIR spectroscopy to investigate the hypothalamus tissues of fatal hypothermic, fatal hyperthermic, and normothermic rats to determine forensically significant related biomarkers. FTIR-differentiated profiles revealed that the spectral variations in the lipid (3100 − 2800 cm^−1^), amide I and II protein bands (1700 − 1500 cm^−1^), and carbohydrate and nucleic acid area (1200 − 900 cm^−1^) components are highly different for hypothalamuses after exposure to fatal hypothermic, hyperthermic, and normothermic conditions. Briefly, in both the fatal hyperthermia and hypothermia groups, the authors obtained higher total lipid amounts; lower unsaturated lipids; alterations of the secondary structures of regular proteins; and different concentration of total proteins, nucleic acids, and carbohydrates. The results validate the potential of FTIR spectroscopy for biochemical determination of fatal hyperthermia and hypothermia hypothalamus tissues as a postmortem diagnostic feature [203].

## 3. Future Perspectives: Framing in a Broader Vision of Health Infrastructure and Policies

The role of vibrational spectroscopy in clinical practice has been boosted tremendously in the recent years due to the cumulated features, namely development of high-tech instruments, efficient data assessment software, nondestructive nature, high resolution, and ability to directly probe biochemical changes without the addition of stains or contrast agents.

Although the in vivo presented studies had positive results, nearly all reports have low statistical power with low reproducibility, with constraints given by the small sample sizes. Therefore, a step change is needed to ensure sufficiently large numbers of animal models to ensure first the accuracy of the results and then to recruit adequate number of patients in order to build diagnostic models able to describe the majority of the variance in the population of interest as well as to test them. From our point of view, a mandatory innovative step that needs to be considered will be the development of standardized disease-spectral databases specific for each type of sample and pathogenesis. This spectral collection will correlate preclinical and clinical research expertise with data technology components aiming to enable the real applicability for health care providers. Most importantly, the designed solution will enhance personalized medicine components and would be made available to all vibrational spectroscopy specialists.

The combination of vibrational spectroscopy with a number of complementary techniques made on the same sample, such as X-ray fluorescence microscopy, histological stains, and immunofluorescence procedures, provides a valuable clinical methodology, acknowledging the comparison of the chemical composition of samples with the distribution of trace elements [19]. Even if vibrational spectroscopy limits the analysis to samples attained by autopsy or biopsy, could it be possible to correlate the standard imaging techniques such as PET, MRI, and CT performed on living patients for early diagnosis and effective disease prognosis?

However, the vibrational spectroscopy still needs further developments from several points of view. Since the clinical applicability is currently a restrictive one, vibrational spectroscopy is considered a ‘’research purpose only methodology” or just a complementary or discriminatory method in diagnosis that requires certification by other techniques, therefore a milestone for public health. Given its contextual utility and relatively low costs, added to the non-negligible benefits of noninvasiveness and minimum supply necessities, this method should be integrated in a more complex vision by policy makers.

Research facilities should be built next to large clinical spaces to increase and broaden the databases and research for the epidemiology. These will feed health policies for major pathologies (a very wide range of cancers, neurological pathologies, gastroenterology, etc.) thus implicitly, eventually, improving the burden problem of the medical system. Furthermore, future studies performed in university centres that may involve large and varied epidemiological samples of patients can become scientific evidence targeting the applicability of vibrational spectroscopy in clinical space as a clear diagnostic method. Also, in a multidisciplinary approach (specific to the centres of excellence in the university environment), vibrational spectroscopy can be used as a tool in the methodologies for calculating risk scores based on socioeconomic factors. Together with biomedical understanding, effective development of practical protocols for point-of-care analysis can be developed, depending on how the new information and the added value offered by vibrational spectroscopic techniques will be integrated into the clinical practice [200].

As a result, we perceive vibrational spectroscopy development as a fashionable topic for health policies in the present and in the future. However, a vast array of potential applications continues to be assessed while others need further technological developments [200], for example, endoscopic disease detection in hollow organs, detection of pathogens in urine samples, detection of pathogens in blood samples, detection of pathogens in other body fluids, identifying antibiotic resistance, vibrational spectroscopy utility in pharmaceutical analysis, and recently developed infrared and Raman spectroscopic instrumentation to whole-organism analysis [204]. In this regard, further new steps are necessary involving economics and practical technology translation from the proof of concept studies.

Progress in artificial intelligence and hardware opens some new opportunities for spectroscopy that are applicable to human health, leveraging its high dimensional analytic capability. In a self-propelled circle, the need for new computational algorithms, new quantitative data, and interplay between practical technology and fundamental research evolution will provide a more efficient design and an integrated analytical system. Also, survey studies that can detect the dynamics of phenomena will help in time to the success of this method if integrated into such an abovementioned framework [200,205].

Taken into consideration all these anticipated advances, will FTIR/Raman spectroscopy become a valuable tool for routine on-site analysis and “point-of-care” applications in near future?

## 4. Conclusions

Impressive progress has been made towards implementing vibrational spectroscopy techniques in the biomedical field and designing assays suitable for point-of-care applications in recent years. The importance of each step for a complete infrared analysis is increasingly appreciated and more studies are published addressing the complete chain of analysis.

Taking together advantages and drawbacks, vibrational spectroscopy techniques could provide the “second opinion” in diagnostic but can also specify new means of understanding disease progression and risk in the future.

## Figures and Tables

**Table 1:** Typical Raman and FTIR band allocations in atherosclerosis plaque.

Raman Peak, cm^−1^	Assignment	Observations	FTIR Peak, cm^−1^	Assignment	Observations
2885, 1674	C–H stretching and bending	Cholesteryl esters and cholesterol [81,82]	3500, 3100	Amide A, B	Proteins [79,80]
1740	C=O stretching		3005	Unsaturated aliphatic compounds	Cholesteryl oleate and linoleate [58,73,74]
1443	C=C stretching		2800–3000	CH_2_/CH_3_	Higher absorbance for lipids than for proteins [58,73,74]
704	Vibration of steroid rings		1710–1750	C=O stretching	Lipids [58,73,74]
1660, 1244	Amide I, III	Proteins [74,81]	1730	C=O	Marker for lipids, cholesteryl esters and triglycerides [58,73,74]
1004	Phenylalanine		1718–1487	Alterations of the protein’s secondary structure [79,80]	
1580, 1130, 750	Heme	Hb [74,81]	1652, 1539, 1236	Amide II and III	Proteins [79,80]
1070–1080	Phosphate stretching	Hydroxyapatite [74,81]			
964	Stretching vibrations of ν(PO_4_)	Calcification [74,81]	1080–1100 and 600	Hydroxyapatite [79,80]	
			1058	C–O	Cholesterol alone [58,73,74]

**Table 2:** Raman wavenumbers of diabetic Sprague Dawley rat blood.

Raman Bands, cm^−1^			Assignments	Observations
Leucine	Isoleucine	Diabetic Blood		
913	907	-	C–C and C–N stretching in leucine and isoleucine	1125, 1395, and 1585 cm^−1^ were considered peaks for diabetes type 2 [108,109,110,111,112,113]
-	-	926	C–O and C–C stretch in glucose	
1106	1108	1108	C–C and C–N stretching in leucine and isoleucine; C–OH and C–O–H stretch in glucose	
-	-	1125	C–OH and C–O-H stretch in glucose	
1236	1248	1248	CH_2_ torsion in leucine and isoleucine C–O–H deformation in leucine	
1302	-	1302	CH deformation in leucine	
1395	-	1395	CH and CH_3_ bending; CH_3_ deformation in leucine	
-	1437	1437	Asymmetric rocking, symmetric bending of C atoms in isoleucine	
-	1585	1585	-	

**Table 3:** Typical FTIR band assignments of an adipose tissue.

FTIR Bands, cm^−1^	Assignment	Observations
3290	N–H (Amide A) and OH symmetric stretching -	Proteins and small input of polysaccharides, carbohydrates and water [115]
3006	CH stretching vibration -	Unsaturated lipids, cholesterol esters [116]
2924, 2854	CH_2_ anti-symmetric and symmetric stretching	Lipids with proteins, carbohydrates, nucleic acids effect [116]
1744	Carbonyl C–O stretch	Triglycerides [116]
1654	Amide I	Protein C–O stretching [115]
1547	Amide II (C–N stretch, protein N–H bend)	Proteins [115]
1469	CH_2_ bending	Acyl chains of lipids [115]
1375	C–N stretching	[115]
1238	Asymmetric PO_2_^−^ stretching	[115]
1164	C–O stretching	Found in normal tissue [115]
1100	Stretching PO_2_^−^ symmetric (phosphate II)	[115]

**Table 4:** FTIR wavenumbers selected to classify the cervical lesions using GA-LDA/QDA model [124].

FTIR Bands, cm^−1^			Assignment	Observations
NILM vs. SIL	NILM vs. LSIL	NILM vs. HSIL		
1747	-	1758	C=O stretching vibrations	Lipids
1724	1724	1729	C=O stretching vibrations	aldehydes
1631	-	1639	C=O stretching vibration; C-N bond stretching	Amide I group coupled with N–H bending
1539	-	1531	C–N stretching and N–H deformation	Amide II
-	1334	1342	Amide III	Proteins
1454	1461	1467	CH_3_ and CH_2_ deformations	Lipids and proteins
1400	-	-	CH_3_	Lipids and proteins
-	960	968	C–H bending	
1219	1221	-	Asymmetric stretching vibrations of phosphate	
1080	1089	-	Symmetric stretching vibrations of phosphate	
1155	-	-	C–O	Carbohydrates
-	-	1043	OH stretching coupled with bending	Glycogen band
-	-	1063	CO–O–C symmetric stretching	Phospholipids and cholesterol esters

**Table 5:** The major bands assignments of an ectocervical cell.

FTIR Bands, cm^−1^	Assignment	Observations
3000 − 2800	C–H stretching of methyl/methylene	Lipids [126]
1735	CO–O–C ester carbonyl stretching vibration	
1665 *; 1650–1655 ** and 1635 ***	Amide I (C=O stretching) coupled with N–H in-plane bending	Peptide moiety (* random coil and β-turns; ** α-helical structures; *** β-pleated structures) [126]
1544	C–N stretching and N–H in-plane bending	Amide II
1400–1450	C–H bending	Lipids and proteins
1305	Amide III	Proteins, aliphatic amino acids [131]
1244 * and 1225 **	Asymmetric stretching vibrations of phosphate	Nucleic acid phosphodiester backbone (* α-DNA, ** β-DNA) [132]
1080	Symmetric stretching vibrations of phosphate	Stronger hydrated tissues and cells [133,134]
1055, 1080, and 1150	C–O stretching bands	Glycogen moiety

**Table 6:** The major bands assignments provided in GI cancers.

Condition	FTIR Peak, cm^−1^		Assignment	Observations
	Normal	Cancer		
Oral cancer	1030	1024 OLK or 1025 OSF	C−O Stretching Coupled with C−O bending	Superficial Layer rich in Glycogen [145,146]
Colorectal cancer	3256	3261	N−H and OH stretching vibrations -	Higher intensity of protein and water for malignant tissues [151]
	1647	1641	Amide I	Large amount of mucus for colon adenocarcinoma [151]
	1547	1544	Amide II	
	1093	1084	PO_2_ group of nucleic acids	Endless replication of DNA in cancerous cells [151]
Gastric cancer	1646	1641/1640/1642	Amide I	(Malign/chronic atrophic/superficial gastritis) [152,153]
	1553	1549/1547/1546	Amide II	
	1317	1313/1306/1316	Amide III, symmetric stretch	

**Table 7:** The main structural shifts observed in IR and Raman spectra of different skin lesions.

Characteristic Bands, cm^−1^		Assignments	Observations	
FTIR	2800–3000	CH_2_	Lipids; BCC tumour cells predominantly [169]	
	1740	Ester and acyl	Lipids; increased amount in BCC tumour cells [170]	
	1650	Amide I	Proteins; variations of the amide I/amide II intensity ratio [170,171]	
	1480–1575	Amide II	Proteins [170]	
	1235–1245	Amide III	Proteins; the amide III and DNA spectral features are modified and enhanced with progression to malignancy [170]	
	980, 1080 and 1240	Nucleic acids: ribose, phosphate	Increased intensity in all tumour types; most intense in BCC; 1080 cm^−1^ shoulder in MM and SCC [170]	
Raman	1420-1450	CH_2_	Lipids in BCC (scissoring vibration) [172]	
	1300	–(CH_2_)_n_–	BCC (in-phase twist vibration) [172]	
NIR-FT Raman	1661	Amide I	Proteins; variations in intensity (MM, PN) [173,174]	
	1451	CH_2_ and CH_3_	Proteins and lipids; wide signal for MM, BCC and SK [174]	
	1309	CH_2_	Lipids; increased intensity (MM, BCC, SK) [173,174]	
	1271	Amide III	Proteins; Decreased intensity (BCC, SCC, SK) [173,174]	
	1247	PO_2_^−^	Nucleic acids and phospholipids	Decrease in SK, BCC [174]
	1080			Increase in SK, SCC [172]
	939	C–C	Proline and valine from proteins and lipids; decrease in BCC MM and SK [174]	

**Table 8:** Distribution of typical absorption bands acknowledged in brain tissues.

Condition	Peak, cm^−1^	Assignment	Observations
Normal mouse white matter	2927, 1469	CH_2_	High concentration of long-chain fatty acids in myelin [19,178]
	1740	C=O	Lipid content
	1550	Amide II	Cerebrum
	1235	P=O	Phospholipids (25.2%)
	1085	OH–C–H	Galactose
Krabbe’s disease	2919	CH_2_	Psychosine accumulation [179]
Normal mouse brain	2956, 2922, 2871, 2851	CH_3_, CH_2_	Strong asymmetric and weak symmetric stretching [184]
	1630, 1640/1658 and 1652	Amide I	α-helical protein secondary structure in neuropil and neuron
Alzheimer’s disease	1623	Amide I	Dense plaque cores of TgCRND8 mice;
	1080 and 1230	C–H	Increased phospholipids
Human grey matter-Normal	1650–1656	Amide I	α-helical conformation [181,183]
	1542	Amide II	
Alzheimer’s disease	1632-1634	Amide I	β-amyloid structure [181,183]
	1540	Amide II	
Normal human substantia nigra of brain	3300, 3080	N–H	Protein [191]
	2960, 2930, 2850, 1460, 1380	CH_3_, CH_2_	Lipids
	1656, 1633	Amide I	Proteins with α-helical structures
	1545	Amide II	
	1300	Amide III	Proteins
	1170	CO–O–C	Lipids
	1085	PO_2_^−^	Nucleic aids
Parkinson’s disease	2930, 2850	CH_2_	Higher intensity [88,186]
	1643, 1682, 1662	Amide I	α-synuclein (β-sheet and β-turn band) [186]
	1236, 1086	PO_2_^−^	Significant intensity decrease [186]
	1173	–CO–O–C	Higher intensity [186]
Normal central nervous system	1735	C=O	Lipids and fatty acids [192,193]
	1690, 1650, 1635	Amide I	β-sheet and α-helix protein secondary structure [194,195]
	1560	C–N	Proteins [196]
	1235, 1080	PO_2_^−^	Phosphodiester and nucleic acids backbone (RNA and DNA) [197]
	965	P–O–C	Nucleic acids (DNA and RNA) [198]
Multiple sclerosis	1690, 1635	Amide I	Controlled by anti-parallel β-pleated and β-pleated sheet constituents [187]
Normal human cerebrospinal fluid	3010	C=CH	Unsaturated lipids [189]
	2920 and 2850	CH2	Long hydrocarbon chains in lipids [189]
	1730	C=O	Proteins [189]
	1657	Amide I	
	1546	Amide II	Lipids [189]
CIS, TCIS, RRMS	1732	C=O	Significant increase in carbonyl amount [189]
	795	Guanine C3′-endo/syn conformation in the Z-DNA [189]	
